# How do case presentation teaching methods affect learning outcomes?-SNAPPS and the One-Minute preceptor

**DOI:** 10.1186/s12909-016-0531-6

**Published:** 2016-01-13

**Authors:** Masayasu Seki, Junji Otaki, Raoul Breugelmans, Takayuki Komoda, Shizuko Nagata-Kobayashi, Yu Akaishi, Jun Hiramoto, Iwao Ohno, Yoshimi Harada, Yoji Hirayama, Miki Izumi

**Affiliations:** Department of Medical Education, Tokyo Medical University, 6-7-1 Nishi-Shinjuku, Shinjuku-ku, Tokyo 160-0023 Japan; Department of Primary Care and General Medicine, Tokyo Medical University, 6-7-1 Nishi-Shinjuku, Shinjuku-ku, Tokyo 160-0023 Japan; Department of General Internal Medicine, Jikei University School of Medicine, 3-25-8 Nishi-Shinbashi, Minato-ku, Tokyo 105-8461 Japan; Center for Medical Education, Graduate School of Medicine, Hokkaido University, North 15, West 7, Kita-ku, Sapporo, Hokkaido 060-8638 Japan; Educational Institutional Research Center, Tokyo Medical University, 6-7-1 Nishi-Shinjuku, Shinjuku-ku, Tokyo 160-0023 Japan

**Keywords:** SNAPPS, One-Minute Preceptor, Teaching method, Junior resident, Case presentation

## Abstract

**Background:**

Various techniques have been developed to enable preceptors to teach residents effectively in outpatient settings to promote active learning, including SNAPPS and the One-Minute Preceptor (OMP). This study aimed to ascertain the differences between SNAPPS and the OMP in case presentation content and learner evaluation when used to teach residents about case presentation.

**Methods:**

From 2011 to 2013, participants were 71 junior clinical residents employed in two hospitals for clinical training. They were randomly allocated to two groups, one using SNAPPS and the other the OMP. From recorded discussions, the “differential diagnoses”, “questions and uncertainties”, “treatment plans”, and “learning issues” were counted. Also, a self-evaluation form was distributed at the end of the study to evaluate the residents’ satisfaction with the case presentation.

**Results:**

Members of the SNAPPS group used significantly more meaning units related to questions and uncertainties compared with those of the OMP group (*P* < 0.001). Self-evaluation sheets revealed that members of the SNAPPS group had significantly higher positive responses than those of the OMP group in terms of the following evaluations: “It was easy to bring up questions and uncertainties” (*P* = 0.046), “It was easy to present the case efficiently” (*P* = 0.002), “It was easy to present the case in the sequence given” (*P* = 0.029), and “I was able to give an in-depth case presentation” (*P* = 0.005).

**Conclusions:**

SNAPPS may induce more meaning units related to questions and uncertainties and give more satisfaction to residents than the OMP.

**Electronic supplementary material:**

The online version of this article (doi:10.1186/s12909-016-0531-6) contains supplementary material, which is available to authorized users.

## Background

Residents are required to report the clinical details of inpatients and outpatients to preceptors as a basic medical competency. As learners, they must also have the ability to connect these clinical details actively to their subsequent studies. Preceptors are required to teach residents how to improve these abilities.

Some techniques have been developed for preceptors to teach residents effectively in outpatient clinic settings where time is limited. Examples include the One-Minute Preceptor (OMP), proposed in 1992, and SNAPPS, proposed in 2003 (Table 1) [1–3]. The OMP provides brief and effective intervention for learners during instruction in outpatient treatment. SNAPPS, a mnemonic consisting of six steps (Table 1), promotes concise case presentations by having learners summarize actual reports and express their ideas and clinical reasoning.

**Table 1:** SNAPPS [1] and One-Minute Preceptor (Six Microskills) [2]

SNAPPS	
1.	Summarize briefly the history and findings
2.	Narrow the differential to 2–3 relevant possibilities
3.	Analyze the differential by comparing and contrasting the possibilities
4.	Probe the preceptor by asking questions about uncertainties, difficulties, or alternative approaches
5.	Plan management for the patient’s medical issues
6.	Select a case-related issue for self-directed learning
One-Minute Preceptor (Six Microskills)	
1.	Get a commitment
2.	Probe for supporting evidence
3.	Teach general rules
4.	Reinforce what was right
5.	Correct mistakes
6.	Identify next learning steps

SNAPPS and the OMP are both used in clinical settings and the differences between both methods have been discussed [3]. However, to the best of our knowledge, no reports have compared experimentally these two methods using the content of case presentations for the same case evaluated by learners. In Japan, few medical schools provide structured instruction in case presentation during bed side learning. Hence, we think Japanese that junior residents need to continuously receive guidance for case presentation. Our study aim was to assess the differences between SNAPPS and the OMP in terms of case presentation content and learner evaluation when these techniques are used to teach Japanese residents about case presentation.

## Methods

### Study design

**Fig. 1:**
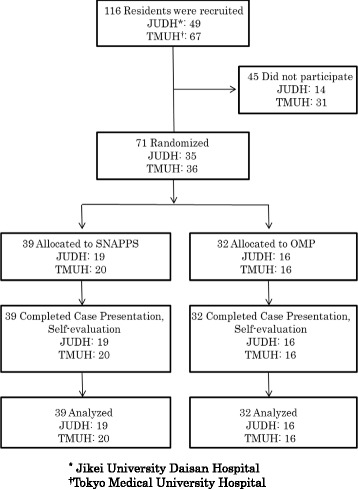
Flowchart of resident case presentation teaching methods study, 2013

This was a comparative study involving a simulated patient case. A single simulated case was used to eliminate the case influences on educational method effects. Residents were randomly allocated to two groups, one taught about case presentation with SNAPPS and the other with the OMP (Fig. 1). The groups were then compared in terms of the self-evaluations and content of the discussion during instruction of each resident. One individual (M.S.) served as the sole study preceptor. He had not routinely used either SNAPPS or the OMP. To prepare for this research, he reviewed these models in a pilot study.

At each hospital, residents were randomly allocated to two groups on the basis of a random number list. One group was taught about case presentation with SNAPPS and the other with the OMP.

### Setting and participants

The potential participants in this study were 116 junior residents who underwent compulsory basic clinical training at Jikei University Daisan Hospital (JUDH) or Tokyo Medical University Hospital (TMUH) between September 2011 and January 2013 (49 at JUDH, and 67 at TMUH) and who were requested to cooperate with the project. These potential participants had passed the National Medical Licensing Examination and were employed as junior clinical residents in post-graduate year (PGY) 1 or 2. Both university hospitals were approved by the Ministry of Health, Labour and Welfare as hospitals for training clinical residents.

### Teaching methods

The OMP started as a five-step microskills teaching model to which a sixth step—“identify next learning steps”—was later added [3–5]. Our study used the OMP six microskills model to make the conditions for comparative investigation as close as possible because SNAPPS includes the step, “probe the preceptor by asking questions about uncertainties, difficulties, or alternative approaches”.

### Resources to explain teaching methods

In SNAPPS, learners are required to understand the six-step framework before presenting cases. Therefore, we produced a Japanese-language video to explain it to residents. This was created by adding Japanese captions to a SNAPPS explanatory video [6] that is publicly available on the Internet, with the permission of its makers. Because the OMP is widely understood and used by preceptors, no prior explanation to learners is usually required. However, to make the conditions between the two trial groups as close as possible, we prepared written documentation for residents in the OMP group and explained it for a length of time comparable with the length of the SNAPPS video (6 min).

### Case

The same paper patient, complaining of low back pain, was used for both groups (see Additional file 1). When preparing the simulated case, we took account of the symptoms, conditions, and diseases for which residents should have experience, as indicated in the Achievement Targets for Clinical Residency [7] stipulated by the Ministry of Health, Labour and Welfare. In view of the bias due to case influences we created a simulated case based on the common symptoms seen in Japanese hospitals that would require residents to think about the differential diagnoses over multiple organs.

### Self-evaluation form

We distributed self-evaluation sheets to the residents participating in the study at the end of the case presentation and discussion about the simulated case and collected information including their thoughts on the method of instruction that had been used for them. The questions on the self-evaluation sheet (Table 2) were prepared after modification from previous studies [8]. The question format was the five-point Likert scale of 0 = “strongly disagree” to 4 = “strongly agree”.

**Table 2:** Self-Evaluation Form about Case Presentation

1.	It was easy to bring up differential diagnoses
2.	It was easy to bring up questions and uncertainties
3.	It was easy to bring up management plans
4.	It was easy to bring up learning issues
5.	It was easy to present the case efficiently
6.	It was easy to present the case in the sequence given
7	I was able to give an in-depth case presentation

### Interventions

Residents at both hospitals who agreed to participate in the study were randomly allocated to one of the two technique groups. After having been asked to read the documentation for the same simulated case, each resident was required to make a case presentation with the same investigator (M.S.) acting as the preceptor, which was then discussed. SNAPPS was used to instruct one group and the OMP for the other group. Sound recordings of the discussions were made with the consent of the residents concerned. The residents in the SNAPPS group were shown a Japanese version of an explanatory video (described above) in advance. The residents in the OMP group were shown a written explanation of the OMP. For both groups, the explanatory materials were collected before the case presentation. If the participants became confused about the correct sequence during the case presentation or discussion, they were reminded as needed. To avoid the discussion being led by the preceptor’s remarks, if the preceptor was asked by a resident about the patient’s correct diagnosis or the necessary plan in the “probe the preceptor” step of SNAPPS or the “teach general rules” step of the OMP, he would ask the same standard question in both groups: “Let’s list and consider the organs that might be the cause.” We explained this study and the teaching methods to each resident, who then presented and discussed the simulated patient case—all within 30 min. After the session, residents were asked to fill in the self-evaluation sheets.

### Measurements

The recorded discussions were transcribed, and the numbers of meaning units [9] used by the resident that were judged to correspond to “differential diagnoses” (DD), “questions and uncertainties” (QU), “management plans” (MP), and “learning issues” (LI) were counted. Meaning units were extracted from the transcripts by three graduate students. These three graduate students were given an explanation and demonstration of the task by using the transcripts from a preliminary study. They worked independently without consulting with each other to extract words associated with DD, QU, MP, and LI, which they then further analyzed and classified into more detailed categories that were established during the preliminary study. The three students subsequently met to compare their results and discuss any points of difference.

### Statistical analysis

The Mann–Whitney U test was used to test the differences between the two groups in the numbers of words used by the learners related to DD, QU, MP, and LI. Prior to this, the Shapiro–Wilk test was used to confirm that none of the data conformed to a normal distribution. The scores for each of the questions on the self-evaluation sheet were counted and compared using the Mann–Whitney U test. The Chi-square test was used to test for differences in the characteristics of participants. The level of significance was *P* = 0.05 for all tests, and IBM SPSS Statistics version 21 was used for statistical analysis.

## Results

Of the 116 potential participants, 71 (35 at JUDH, 36 at TMUH) agreed to cooperate with and actually participated in the study, with data obtained from all participants (61 % of potential participants). There were no differences in the characteristics (sex, PGY, hospital) of participants between study groups (Table 3).

**Table 3:** Characteristics of Study Participants

	SNAPPS (*n* = 39)	One-minute preceptor (Six Microskills) (*n* = 32)	*P*-value
Sex			0.75
Male (%)	27 (69 %)	21 (66 %)	
Female (%)	12 (31 %)	11 (34 %)	
Experience			0.25
Post-graduate year1 (%)	30 (77 %)	28 (88 %)	
Post-graduate year2 (%)	9 (23 %)	4 (12 %)	
Hospital			0.91
Tokyo Medical University Hospital (%)	20 (51 %)	16 (50 %)	
Jikei University Daisan Hospital (%)	19 (49 %)	16 (50 %)	

**Fig. 2:**
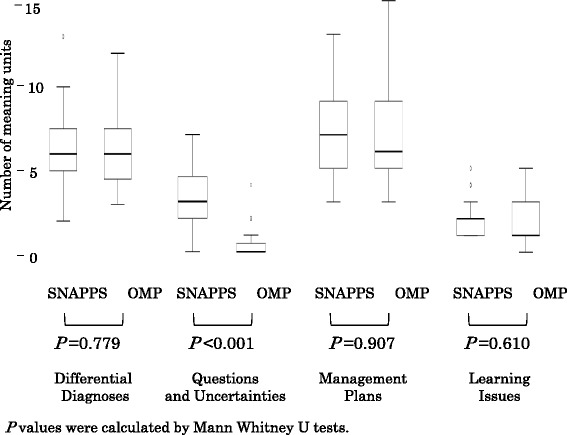
Median and range of the number of residents’ words

Figure 2 shows the numbers of meaning units related to DD, QU, MP, and LI that were used by the residents. Members of the SNAPPS group used significantly more meaning units related to QU than members of the OMP group did (*P* < 0.001).

**Fig. 3:**
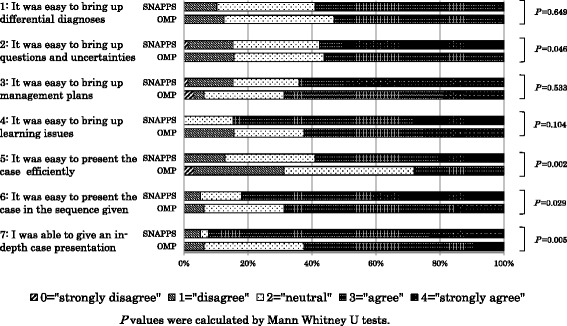
Comparison of residents’ case presentation self-evaluation scores between the SNAPPS and One-Minute Preceptor groups

The self-evaluation sheets revealed significant differences between the SNAPPS and OMP groups in terms of the following evaluations: “It was easy to express questions and uncertainties” (*P* = 0.046), “It was easy to present the case efficiently” (*P* = 0.002), “It was easy to present the case in the sequence given” (*P* = 0.029), and “I was able to give an in-depth case presentation” (*P* = 0.005) (Fig. 3).

## Discussion

Our study investigated the content of discussion and differences in residents’ evaluations of teaching when SNAPPS or the OMP was used to instruct residents on case presentation. Previous studies have found that both techniques are structures that improve the educational process and outcome in outpatient clinics [3, 10].

A teaching model proposed by Wolpaw et al. (Table 1) [2], SNAPPS promotes learning concise case presentations by having learners summarize actual reports and express their ideas and deductions. Learners are required first to understand the six-step process and then to present a case in accordance with the steps. A feature of this procedure is that it focuses not on the questions or explanations of the preceptor, but rather on encouraging learners to raise questions themselves to accelerate problem-solving and autonomous learning. The use of SNAPPS has been reported to increase the number of disorders in the differential diagnosis and to enable learners to establish questions and issues for themselves [11].

The OMP is a five-step microskills teaching model proposed by Neher et al. in 1992 (Table 1) [1]. Its aims are to clarify the knowledge and procedures used to resolve problems and to intervene in areas where knowledge and procedures are lacking. Although the OMP has a five-step structure, as it is used mainly in outpatient clinic teaching to provide effective intervention for learners in a short time period, it has also been described in terms of six microskills with the later addition of “identify the next learning steps” as a sixth step [3–5]. Compared with conventional teaching methods, the OMP is well known to enable more debate on disorders in the differential diagnosis, tests, and characteristic symptoms [12], generates more information in the same amount of instruction time, and results in a higher rate of correct diagnoses by the preceptor [13].

In our study, we observed no statistically significant differences in the number of meaning units related to DD, MP, or LI that were elicited from residents who presented and discussed the case by either SNAPPS or the OMP, but residents in the SNAPPS group used significantly more meaning units related to QU than residents in the OMP group did. SNAPPS is structured to encourage learners to express QU, therefore it may have elicited more QU than instruction using the OMP.

More participants in the SNAPPS group than in the OMP group chose to “strongly agree” to the statement, “It was easy to bring up MP,” but when the responses “strongly agree” and “agree” were combined, they were chosen by more participants in the OMP group. The reason for this sharp divergence of opinion between positive and negative evaluations among members of the SNAPPS group may be that SNAPPS aims to encourage problem-solving and autonomous learning; therefore, some residents may have found it difficult to focus the discussion on MP [1]. The high proportion of positive evaluations from members of the OMP group may stem from the fact that the OMP goal is effectively intervening with learners quickly, which enabled them to focus more readily on MP [2].

Most participants in the SNAPPS group strongly agreed with the statement, “It was easy to bring up LI,” and none chose the response “strongly disagree” or “disagree.” A high proportion of members of the OMP group chose the response “disagree.” The high positive response from members of the SNAPPS group may have been because SNAPPS aims to encourage problem-solving and autonomous learning, making it easy to bring up LI [1]. The divergence of opinion among participants in the OMP group may have been because the OMP’s objective is to explain clinical procedures and intervene in areas where learners are lacking. Therefore, even with the sixth stage of “Identify next learning steps” added to the five microskills, it may still have been difficult to focus the discussion on bringing up LI [2, 4, 5].

Similar proportions of residents in both our study groups chose the responses “agree” and “strongly disagree” for “It was easy to bring up QU,” although no participants in the OMP group chose “strongly agree.” The fact that only SNAPPS has a procedure to elicit QU may contribute to the strong, positive evaluation by members of the SNAPPS group. Also, the number of meaning units related to QU was greater in the SNAPPS group than in the OMP group (Fig. 2).

In terms of residents’ evaluations of the method of instruction used, members of the SNAPPS group evaluated the statements “It was easy to present the case efficiently,” “It was easy to present the case in the sequence given,” and “I was able to give an in-depth case presentation” significantly more highly than did participants in the OMP group. The fact that SNAPPS is a learner-centered model may contribute to this high evaluation [1].

### Limitations

Our study measured the contents of residents’ case presentations and discussions and their evaluations of the method of instruction.

It is necessary to consider whether the outcome of the method can be generalized. Because a simulated case was used in this study, the thought processes involved in bringing up DD, MP, QU, and LI may differ from those in actual clinical settings.

The same preceptor used a simulated case to teach participants in two different institutions, therefore it is unknown whether similar results can be obtained when different doctors use these methods with respect to real patients in clinical settings.

During the explanation of the teaching method to residents before the start of the session, participants in the SNAPPS group watched a video, but participants in the OMP group read a document. The difference in material to explain the teaching method may contribute to differences in learners’ evaluations of the teaching method.

The OMP is used to provide effective intervention for learners in a short time, and in this study, 30 min from explanation to discussion was allotted to make the conditions for the SNAPPS and OMP groups as similar as possible. The distinguishing features of the OMP may therefore have been lost. [2]

It is important to take national and regional cultural differences sufficiently into account when disseminating educational theories [14, 15]. The style of communication in East Asia is sometimes described as “cultural reticence,” which consists of a reluctance to speak and a tendency not to express as much as is known or felt [16]. Further studies are needed to determine whether similar results to ours will be obtained in other countries and regions whose culture and communication differ from Japan’s.

## Conclusions

Comparison of teaching case presentations using SNAPPS or the OMP revealed differences in the content and discussion of case presentations and in residents’ evaluations of the teaching methods. SNAPPS may induce more meaning units related to questions and uncertainties and give more satisfaction to residents than the OMP. Within each teaching method group, there were individual resident differences in the outcome of teaching. For both SNAPPS and the OMP, preceptors require a deep understanding of the teaching method and an ability to teach that considers the characteristics of the learner. Further studies are needed to investigate the extent to which the learner’s characteristics and cultural background affect the case presentation.

### Ethical approval

This study was approved by the respective Institutional Review Boards of JUDH and TMUH.

## Additional file

Additional file 1:
**The simulated case (paper patient).** (DOCX 16 kb)
