# Supplementary material for: How do case presentation teaching methods affect learning outcomes?-SNAPPS and the One-Minute preceptor
Source: BMC Med Educ. 2016 Jan 13;16:12. doi: 10.1186/s12909-016-0531-6 (PMC4712551; doi:10.1186/s12909-016-0531-6)
Supplement: Additional file 1: — The simulated case (paper patient). (DOCX 16 kb) [file 12909_2016_531_MOESM1_ESM.docx]

Additional file: Case

70-year-old man

【Chief complaint】Low back pain

【Present illness】

The patient had felt a vague heaviness in the lumbar region from around 18:00 the previous evening. As the pain was bearable, he did not seek treatment. He ate supper as usual and went to bed, but was woken at around 5 a.m. by low back pain. Initially the pain was too severe for him to move, but by 7 a.m. it had improved somewhat. He was too worried to go to work, and so came to the hospital for an examination. On review of systems, he denied headache, cough, sputum or vomiting. His stool was somewhat soft.

【Past medical history】

Hypertension, Hyperlipidemia (attending another hospital)

Smoking: 5 cigarettes/day until 10 years ago

Alcohol: Beer 350 ml/day

【Medication】

Angiotensin Ⅱ Receptor Blocker, HMG-CoA reductase inhibitor

【Allergies】

None

【Physical findings】

Consciousness: alert, BP: 160/90 mmHg, PR: 90 bpm, RR 18/min, BT: 36.5ºC, SpO2 97% (room air)

Throat: No redness

Neck: No lymphadenopathy

Chest: No murmur, no wheeze or crackles, but respiration shallow

Abdomen: The wall flat and soft, no tender, normal active bowel sounds

Back: Left costovertebral angle tenderness

Extremities: No numbness or paralysis

Skin: No rash
